# Risk factors for latent tuberculosis infection in close contacts of active tuberculosis patients in South Korea: a prospective cohort study

**DOI:** 10.1186/s12879-014-0566-4

**Published:** 2014-11-18

**Authors:** Seung Jun Lee, Seung Hun Lee, You Eun Kim, Yu Ji Cho, Yi Yeong Jeong, Ho Cheol Kim, Jong Deog Lee, Jang Rak Kim, Young Sil Hwang, Hee Jin Kim, Dick Menzies

**Affiliations:** Department of Internal Medicine, College of Medicine, Gyeongsang National University, 90 Chilam-Dong, Jinju, Gyeongnam 660-302 South Korea; Gyeongsang Institute of Health Sciences, Gyeongsang National University, Jinju, South Korea; Department of Preventive Medicine, College of Medicine, Gyeongsang National University, Jinju, South Korea; Korean Institute of Tuberculosis, Seoul, South Korea; Montreal Chest Institute, McGill University, Montreal, Canada

**Keywords:** Tuberculin test, Interferon-gamma release tests, Latent tuberculosis, Independent living, Tuberculosis, pulmonary

## Abstract

**Background:**

The diagnosis and treatment of latent tuberculosis infection (LTBI) have become mandatory to reduce the burden of tuberculosis worldwide. Close contacts of active TB patients are at high risk of both active and LTBI. The aim of this study is to identify the predominant risk factors of contracting LTBI, persons in close contact with TB patients were recruited. This study also aimed to compare the efficacy of the tuberculin skin test (TST) and QuantiFERON^®^-TB GOLD (QFT-G) to diagnose LTBI.

**Methods:**

Close contacts of active pulmonary TB patients visiting a hospital in South Korea were diagnosed for LTBI using TST and/or QFT-G. The association of positive TST and/or QFT-G with the following factors was estimated: age, gender, history of Bacillius Calmette-Guerin (BCG) vaccination, history of pulmonary TB, cohabitation status, the acid-fast bacilli smear status, and presence of cough in source cases.

**Results:**

Of 308 subjects, 38.0% (116/305) were TST positive and 28.6% (59/206) were QFT-G positive. TST positivity was significantly associated with male gender (OR: 1.734; 95% CI: 1.001-3.003, p =0.049), history of pulmonary TB (OR: 4.130; 95% CI: 1.441-11.835, p =0.008) and household contact (OR: 2.130; 95% CI: 1.198-3.786, p =0.01) after adjustment for confounding variables. The degree of concordance between TST and QFT-G was fair (70.4%, κ =0.392).

**Conclusions:**

A prevalence of LTBI among close contacts of active pulmonary TB patients was high, and prior TB history and being a household contact were risk factors of LTBI in the study population.

**Electronic supplementary material:**

The online version of this article (doi:10.1186/s12879-014-0566-4) contains supplementary material, which is available to authorized users.

## Background

According to the report of World Health Organization (WHO), there are about 8.6 million incident cases of tuberculosis (TB) and about 1.3 million deaths from TB annually [1]. A Stop TB Partnership has been established to reduce the TB burden to less than 100,000 cases per year by 2050 by focusing on areas of high TB burden [2]. However, persons in close contacts with TB patients are also at a risk of contracting latent TB infection (LTBI). In 2010, the WHO estimates that nearly 1/3 of the world's population contracted LTBI, and that 10% of these carriers will develop an active TB infection [3]. The vast majority of these patients will become infectious and perpetuate the cycle of morbidity and mortality. Therefore, it is imperative to diagnose LTBI patients early and efficiently to reduce the global burden of TB.

The traditional method to diagnose TB infection is tuberculin skin test (TST). However, this test does not discriminate LTBI patients. The FDA recently approved the use of interferon gamma release assays (IGRAs) such as the QuantiFERON-TB Gold^®^ (QFT-G) assay for the diagnosis of LTBI [4]. In 2011, Korean guidelines stipulate that IGRAs are an alternative method of TST for the diagnosis of LTBI [5]. IGRAs have some advantages over TST. First, IGRAs lack booster effect. Second, BCG vaccination and infection by non-tuberculosis mycobacterium (NTM) has less effect on the IGRA than on the TST. Third, the patients don't need to revisit for reading the results [6]. There are several risk factors known to be associated with developing active TB in close contacts, including malnutrition, untreated LTBI, being a household contact, age under 5 years, acid-fast bacilli (AFB) positivity of source case, concomitant human immunodeficiency virus infection, and immunocompromised status [7],[8]. However, there is limited data about risk factors for LTBI in close contacts.

The present study was conducted to identify the predominant risk factors of contracting the latent form of the disease and to compare the efficiency of the TST and QFT-G to diagnose LTBI subjects in close contacts of active pulmonary TB patients.

## Methods

### Subjects and study design

All data presented in this study were acquired during an international multi-center study (title: A Randomized Clinical Trial Comparing 4RIF vs. 9INH for Treatment of Latent Tuberculosis Infection - Effectiveness, Registration number; NCT00931736). The primary objective of the multi-center study is to compare the effectiveness of two regimens in preventing active tuberculosis. Full details of the original study are provided in the study protocol, see Section 2 in the Additional file 1: Appendix. In this study, close contacts of culture-positive pulmonary TB patients who visited Gyeongsang National University Hospital, Jinju, South Korea, were prospectively enrolled between October 2009 and August 2012. Close contacts are recommended to visit a hospital because screening of close contacts is a national policy project in South Korea. The current study was approved by the Gyeongsang National University Hospital Institutional Review Board (approval number IRB-2009-042). Close contact was defined as exposure to AFB smear-positive and/or culture-positive active pulmonary TB patient during their infectious period for at least 4 hours per week. The duration of exposure was determined by self-reported questionnaire. Household contacts were defined as those living in the same house with source case. Exclusion criterion was active TB identified by chest X-ray. The remaining subjects filled informed consent documents and were tested for LTBI by TST and/or QFT-G. Both tests were conducted on the same day or TST was conducted first. We also documented seven potential risk factors for LTBI: age, gender, history of Bacillus Calmette-Guerin (BCG) vaccination, history of pulmonary TB, living in the same house with a TB patient, the presence of cough, and AFB smear status of the source case. By interviewing enrolled subjects and source patients, age, gender, history of tuberculosis in the close contacts and cough symptom of source case were recorded. History of BCG vaccination was verified by BCG scar. The previous history of TB was identified by self-reported questionnaire. Level of contact was categorized into household or non-household.

### TST and QFT-G

TST was conducted by Mantoux method using purified protein derivative (PPD, Statens Serum Institute; Copenhagen, Denmark). The subjects were given an intradermal injection of Tuberculin (2 units in 0.1 ml) on the forearm. Transverse diameter of induration was measured and recorded in millimeters between 48 and 72 hours. TST was considered positive when induration size was equal to or greater than 5 mm according to the cut-off value of the multi-center study. Induration was read by skilled and trained physicians. QFT-G test (Cellestis Ltd, Carnegie, VIC, Australia) was carried out according to the manufacturer's instructions. Briefly, whole blood was collected and aliquots were incubated for 16 to 24 hours with TB-specific antigens, including early secretory antigenic target 6-kda protein (ESAT-6) and culture filtrate protein 10 (CFP-10). The concentration of released IFN-γ was measured by enzyme-linked immunosorbent assay (ELISA) [9],[10].

### Statistical analysis

Descriptive statistics were used to assess the correlation between the seven potential risk factors and TST and/or QFT-G positivity. Each variable was analyzed using a Pearson's Chi-square test. Risk of having LTBI for each potential risk factor was estimated using multinomial logistic regression. Kappa (κ) statistics were used to measure concordance between TST and QFT-G and classified as follows: κ ≤0 = no agreement; 0 < κ ≤0.2 = poor agreement; 0.2 < κ ≤0.4 = fair agreement; 0.4 < κ ≤0.6 = moderate agreement; 0.6 < κ ≤0.8 = substantial agreement; 0.8 < κ ≤1.0 = optimal agreement. Differences were considered statistically significant if p <0.05. Statistical analyses were performed using SPSS version 18.0 for Window (SPSS Inc, Chicago, Illinois, USA).

## Results

### LTBI in the study population

The review of 129 cases of active TB patients identified a total of 308 close contact individuals (103 males and 205 females) confirmed not to have active TB by chest X-rays (Table 1). Subjects were household or work contacts of patients with confirmed active pulmonary TB. TST was administered and read for 305 subjects of whom 116 (38.0%) had a positive result. QFT-G was carried out in 206 subjects of whom 59 (28.6%) showed a positive result. Both tests were completed in 203 subjects of whom 138 subjects underwent both tests on the same day. QFT-G was conducted later in 65 subjects and median interval between both tests was 3 days. More than 90% of the subjects had a BCG vaccination (94.4% in the TST group, 94.2% in the QFT-G group). A total of 7.2% subjects from the TST group and 7.3% subjects from the QFT-G group had a history of previous active pulmonary TB. AFB staining of smear of source cases was positive in 77.4% in the TST group and 73.8% in the QFT-G group. A total of 53.8% subjects in the TST group and 51.0% subjects in the QFT-G group were household contacts. Presence of cough in source cases was 60.3% in the TST group and 58.7% in the QFT-G group (Table 1). Comorbid conditions included hypertension in 21, diabetes in 8, cardiovascular disease in 7, thyroid disease in 7, chronic liver disease in 5 subjects (data not shown).

**Table 1:** **Baseline characteristics of the study population**

	TST participants	QFT-G participants
	(n = 305)	(n = 206)
Demographics		
Male	103 (33.8%)	66 (32.0%)
Age (Mean ± SD)	44.2 ± 15.1	43.7 ± 14.7
15 - 24	28 (9.2%)	19 (9.2%)
25 - 34	61 (20.0%)	42 (20.4%)
35 - 44	75 (24.6%)	52 (25.2%)
45 - 54	72 (23.6%)	48 (23.3%)
55 - 64	41 (13.4%)	29 (14.1%)
≥ 65	28 (9.2%)	16 (7.8%)
Household contact		
Yes	164 (53.8%)	105 (51.0%)
Test result		
Positive	116 (38.0%)	59 (28.6%)
BCG vaccination scar		
Yes	288 (94.4%)	194 (94.2%)
History of pulmonary TB		
Yes	22 (7.2%)	15 (7.3%)
AFB smear status of source cases		
Positive	236 (77.4%)	152 (73.8%)
Cough of source cases		
Yes	184 (60.3%)	121 (58.7%)

*TB* Tuberculosis, *BCG* Bacillus of Calmette-Guerin, *TST* Tuberculin skin test, *QFT-G* QuantiFERON^®^ TB GOLD.

### Risk factors for LTBI

The three potential risk factors that were not associated with TST- or QFT-positivity were as follows: history of BCG vaccination, cough and AFB smear status of source case (Table 2). Age was associated with TST positivity when stratified into 10-year old, but not with QFT-G positivity. Occurrence of positive TST was significantly higher in males than in females, but there was no significant difference in the occurrence of positive QFT-G between males and females. Both TST- and QFT-positivity were significantly associated with being a household contact. Subjects with history of TB showed significantly higher TST and QFT-G positivity than those without TB (Table 2).

**Table 2:** **Association of seven potential risk factors with TST or QFT-G positivity**

	TST positive, n (%)	p value	QFT-G positive, n (%)	p value
Age		< 0.001		0.063
15 - 24	2 (7.1)		2 (10.5)	
25 - 34	16 (26.2)		6 (14.3)	
35 - 44	39 (52.0)		19 (36.5)	
45 - 54	34 (47.2)		16 (33.3)	
55 - 64	16 (39.0)		10 (34.5)	
≥ 65	9 (32.1)		6 (37.5)	
Gender		0.001		0.176
Male	53 (51.5)		23 (34.8)	
Female	63 (31.2)		36 (25.7)	
BCG vaccination scar		0.430		0.092
Yes	108 (37.5)		53 (27.3)	
No	8 (47.1)		6 (50.0)	
Household contact		0.023		0.033
Yes	72 (43.9)		37 (35.2)	
No	44 (31.2)		22 (21.8)	
History of pulmonary TB		0.001		0.028
Yes	16 (72.7)		8 (53.3)	
No	100 (35.3)		51 (26.7)	
Smear status of source cases		0.139		0.118
Positive	95 (40.3)		48 (31.6)	
Negative	21 (30.4)		11 (20.4)	
Cough of source cases		0.053		0.094
Yes	78 (42.4)		40 (33.1)	
No	38 (31.4)		19 (22.4)	

*TB* Tuberculosis, *BCG* Bacillus of Calmette-Guerin, *TST* Tuberculin skin test, *QFT-G* QuantiFERON^®^-TB GOLD.

Multivariate logistic regression was performed with either TST- or QFT-positivity as the dependent variable and the seven risk factors as independent variables (Table 3). Male gender, history of pulmonary TB and household contact significantly increased odds ratio of TST positivity (Table 3). There was definitely no gender difference in QFT-G positivity, but there was a trend toward QFT-G positivity associated with history of pulmonary TB and household contact. These results suggest that prior TB history and being a household contact are the predominant risk factors for LTBI.

**Table 3:** **Multivariate analysis between seven risk factors and TST or QFT-G positivity**

	TST positivity		QFT-G positivity	
	Odds ratio (95% CI)	p value	Odds ratio (95% CI)	p value
Gender (male)	1.734 (1.001-3.003)	0.049	0.982 (0.476-2.025)	0.961
Age				
15 - 24	1		1	
25 - 34	6.373 (1.273-31.909)	0.024	1.495 (0.267-8.360)	0.647
35 - 44	13.769 (2.865-66.172)	0.001	4.103 (0.809-20.820)	0.088
45 - 54	10.552 (2.221-50.141)	0.003	3.081 (0.607-15.625)	0.174
55 - 64	6.597 (1.307-33.284)	0.022	3.070 (0.555-16.975)	0.199
≥ 65	2.089 (0.284-15.379)	0.470	1.340 (0.134-13.355)	0.803
BCG vaccination scar (yes)	0.335 (0.067-1.681)	0.184	0.361 (0.055-2.370)	0.289
History of pulmonary TB (yes)	4.130 (1.441-11.835)	0.008	2.561 (0.819-8.002)	0.106
Household contact (yes)	2.130 (1.198-3.786)	0.010	1.911 (0.920-3.971)	0.083
Smear status of source case (positive)	1.460 (0.745-2.864)	0.271	1.947 (0.831-4.564)	0.125
Cough of source case (yes)	1.324 (0.753-2.328)	0.329	1.167 (0.576-2.362)	0.669

*TB* Tuberculosis, *BCG* Bacillus of Calmette-Guerin, *TST* Tuberculin skin test, *QFT-G* QuantiFERON^®^-TB GOLD, *CI* Confidence interval.

### Concordance between TST and QFT-G

Among total of 308 participants, 305 subjects performed TST and 206 subjects performed QFT-G testing. Two-hundred-three subjects completed both TST and QFT-G testing. Among them, 46 subjects were positive for both TST and QFT-G, 49 subjects were TST positive but QFT-G negative, 11 subjects were QFT-G positive but TST negative, and 97 subjects were negative for both tests. Concordance rate of two tests was 70.4%. The kappa value showed fair agreement (κ = 0.392), but showed a significant correlation between two tests (p <0.001, Table 4).

**Table 4:** **Correlation between TST and QFT-G (n =203)**

		QFT-G				
		Positive	Negative	κ	p value	Agreement, %
TST	Positive	46	49			
	Negative	11	97	0.392	<0.001	70.4

*TST* Tuberculin skin test, *QFT-G* QuantiFERON^®^-TB GOLD.

## Discussion

This study showed that positive rate of TST and QFT-G is high in those who are in close contact with active pulmonary TB patients. History of TB and household contact were significantly associated with the risk of TST positivity and concordance between TST and QFT-G was 70.4%.

The prevalence of LTBI is expected to be high in close contacts of active TB patients. The prevalence of LTBI in close contacts was reported to be highest among many risk groups for LTBI, including close contacts, foreign-born people, homeless people, injection drug users, and prisoners [11]. Furthermore, close contact is one of the common risk factor for progression from LTBI to active disease [11]. In the present study, the positive rates of TST and QFT-G were 38.0% and 28.6% respectively, in South Korean population. Marks et al. reported that a prevalence of LTBI is about 36% in close contacts of persons with infectious TB as assessed by TST in a study conducted in the United States [12]. Meanwhile, the prevalence of LTBI in household contacts in countries with a high burden of TB is diversely reported from 27% to 93%, again based on TST [13]. The positive rate of QFT-G in contact investigation for TB was 30.2% in Germany and 19% in Taiwan [14],[15]. Given that South Korea has an intermediate burden of TB cases and almost every child receives BCG vaccination at birth, the positive rates of both tests in this study, especially of QFT-G, were considerably high. In a previous multi-center study conducted in South Korea, the positive rate of QFT-G in health care workers was 17.2% and that of TST was 36.7% [16]. Because QFT-G is not affected by the previous BCG vaccination and most of the NTM infection, high positive rate of QFT-G in this study reconfirms the incidence of LTBI in close contacts of active TB patients is quite high in South Korean population.

Increasing age was significantly associated with TST positivity, but not with QFT-G positivity in the current study. Li et al. reported that prevalence of TST positivity significantly increased with age [17]. Furthermore, according to the reports of Pareek et al. and Shanaube et al., the result of QFT-G as well as that of TST was associated with increasing age [18],[19]. It is uncertain whether increased age is the risk factor of contracting LTBI or whether increased cumulative exposure to *Mycobacterium tuberculosis* and/or NTM as people grow older increases positivity of both tests.

Active TB is reported to be more prevalent in men than in women, but reasons for this phenomenon are uncertain due to the lack of studies about gender differences in patients with TB. Gender differences in patients with LTBI were also not studied much. In our study, male gender significantly increased relative risk of TST positivity but not of QFT-G positivity. More data will be needed to clarify the influence of gender in the incidence of LTBI.

The occurrence of LTBI in close contacts with previous pulmonary TB history was significantly high in this study. We aimed to assess the association between previous history of pulmonary TB and the risk of contracting LTBI because the impact of previous history of pulmonary TB on the LTBI had not been well studied. TST and QFT-G are methods to measure lasting adaptive immune responses to *Mycobacterium tuberculosis* rather than to identify LTBI directly [20]. Direct identification of LTBI was impossible until recently. Although IGRA is a officially approved test for the diagnosis of LTBI, Kim et al. reported that IGRA has a limited role in the diagnosis of TB infection in individuals with a history of TB [21]. Thus, it is uncertain whether positive results of TST and QFT-G depend on the presence of living mycobacteria or persistent immune responses of previous TB infection. Nevertheless, close contacts of active TB patients are at a high risk of infection, and QFT-G is suggested to detect INF-γ released by effector T-cells rather than memory T-cells within a short period of incubation [22],[23]. It is therefore mandatory to study the effects of previous TB history on LTBI, including through clinical trials, and to understand the molecular basis of T-cell responses.

Household contact increased the risk of TST- and IGRA-positivity in previous studies [24],[25]. Likewise, the result of current study showed that household contact significantly increased relative risk of being TST positive and QFT-G was tend to be more positive in household contacts. It is therefore reasonable to pay particular attention to household contacts during the contact investigation of active TB.

The agreement and kappa values of concordance of TST and IGRA have been reported widely in TB contacts [9],[15],[26]. The common outcome of those studies is that BCG vaccination lowered the agreement between the two tests. In one study performed in Denmark, in which about two-thirds of enrolled subjects were not vaccinated with BCG, the agreement between TST and QFT-G was 94% (κ = 0.866) [9]. In contrast, most of the people in Korea receive BCG vaccine, and the agreement of two tests in this study was much lower (70.4%, κ = 0.392). In this study, only 11 of 203 subjects who received both tests were not vaccinated with BCG. Thus, subgroup analysis for non-BCG vaccinated people did not show significant results (data not shown). Additionally, high rate of BCG vaccination lowered the statistical power to assess the impact of BCG vaccination on TST or QFT-G. In this study, 11 subjects were QFT-G positive but TST negative. The result of QFT-G might be false positive even though we could not verify the baseline result of QFT-G before contact to active TB patients. Performing QFT-G test repeatedly will be helpful to clarify this issue.

While designing this study, we assumed that smear status and presence of cough in source cases might be important factors influencing the occurrence of LTBI. However, our results show that none of these were risk factors. Therefore, the limitations of this study are as follows. First, the number of enrolled subjects is relatively small because people in Korea are not aware of the concept of LTBI and the importance of screening contacts of TB patients. The knowledge about LTBI needs to be spread among the Korean people. Second, of the 308 enrolled subjects, only 203 subjects were willing to take both TST and QFT-G tests, because we gave them an option of choosing between TST and/or QFT-G according to the Korean guidelines. Both single screening strategy using TST or IGRA and dual screening strategy using TST and subsequent IGRA were recommended in the Korean guidelines. Third, in 65 among 203 subjects who took both tests, QFT-G was conducted later than TST and in 28 among those 65 subjects, the interval between two tests was over 4 days. Van Zyl-Smit et al. have reported that a booster effect of PPD was evident by day 7 post-TST, but not by day 3 [27]. The booster effect of PPD might have caused false-positive results of QFT-G in some of the subjects in this study. Fourth, the level of contact was not categorized in depth. In particular, in subjects who were a household contact, whether they lived in the same room or not would be an important factor influencing their LTBI risk.

## Conclusions

LTBI is prevalent in close contacts of active pulmonary TB patients in a South Korean population, and history of pulmonary TB and being a household contact are predominant risk factors of contracting LTBI in this study.

## Authors' contributions

HCK and SJL are guarantors of the manuscript. H.C. Kim: contributed as the corresponding author, reviewing all data, and revising the manuscript. SJL: contributed as the primary author, reviewing all data, and writing the manuscript. YEK: contributed to the preparation of the manuscript, and read and approved the final manuscript. YJC: contributed to the preparation of the manuscript, and read and approved the final manuscript. YYJ: contributed to the preparation of the manuscript, and read and approved the final manuscript. JDL: contributed to the preparation of the manuscript, and read and approved the final manuscript. JRK: contributed to the preparation of the manuscript, and read and approved the final manuscript. YSH: contributed to the preparation of the manuscript, and read and approved the final manuscript. HJK: contributed to the preparation of the manuscript, and read and approved the final manuscript. DM: contributed to the preparation of the manuscript, and read and approved the final manuscript. All authors read and approved the final manuscript.

## Additional file

## Electronic supplementary material

Additional file 1: A randomized clinical trial of 4 months Rifampin vs. 9 months Isoniazid for latent TB infection. - Phase 3 effectiveness. (DOC 826 KB)

## Abbreviations

WHO: World health organization
TB: Tuberculosis
LTBI: Latent TB infection
TST: Tuberculin skin test
IGRA: Interferon gamma release assay
QFT-G: QuantiFERON-TB^®^ Gold
NTM: Non-tuberculosis mycobacterium
AFB: Acid-fast bacilli
BCG: Bacillus calmette-guerin
PPD: Purified protein derivative
