# Supplementary material for: Risk factors for latent tuberculosis infection in close contacts of active tuberculosis patients in South Korea: a prospective cohort study
Source: BMC Infect Dis. 2014 Nov 18;14:566. doi: 10.1186/s12879-014-0566-4 (PMC4237765; doi:10.1186/s12879-014-0566-4)
Supplement: Supplementary file 1 — Additional file 1: A randomized clinical trial of 4 months Rifampin vs. 9 months Isoniazid for latent TB infection. - Phase 3 effectiveness. (DOC 826 KB) [file 12879_2014_566_MOESM1_ESM.doc]

A randomized clinical trial of 4 months Rifampin vs. 9 months Isoniazid

for latent TB infection. – Phase 3 effectiveness

**SECTION 1 THE NEED FOR A TRIAL**

**1.1. What is the problem being addressed?**

**1.1.1 Importance of tuberculosis (why study TB?)**

*1.1.1.1 Globally:* As a disease, tuberculosis (TB) is unique in that it is wholly preventable and treatable, yet on a global scale, incidence and mortality continue to rise ^1^. The World Health Organization (WHO) has estimated that there are between 8 and 9 million new cases each year ^2^, that 200 million persons alive today will develop active TB during their lifetime ^2^, and that 30 million will die from TB over the next decade ^2^ - equivalent to the population of Canada. TB remains the world’s most important infectious cause of morbidity and mortality among adults, yet remarkably, many consider this disease to be of little current significance or importance ^3^. In many industrialized countries, after decades of decline, incidence of tuberculosis increased in the 1980's. In most, with greater investment in TB control, rates have since levelled off or declined somewhat ^4, 5^ although in some, such as Britain ^6^, incidence continues to increase.

*1.1.1.2 TB in Canada (why study TB in Canada?):* Incidence of active TB in Canada declined steadily from the beginning of the 20th century until the mid 1980's. Since then, the number of new cases reported each year has remained largely unchanged. In 2006, overall incidence was 5 new active cases per 100,000 population ^7^, but rates were substantially higher in certain populations and regions, because TB remains a disease of poor and marginalised populations ^8-10^. Incidence among aboriginals ranges from 25 to 50 per 100,000 compared to less than 3 per 100,000 among non-aboriginal Canadian-born ^7^. Rates among the foreign-born are three times higher than the national average ^7^; 90% ^11-14^ of cases among foreign-born arise because of reactivation of dormant TB infection acquired before immigration, in their countries of origin ^15-17^.

**1.1.2 Pathogenesis of tuberculosis (why treat latent TB infection?)**

TB infection is transmitted by the airborne route from patients with active pulmonary TB disease. In more than 95% of individuals who acquire primary infection, there is no clinical illness and the TB bacilli enter a latent or dormant state; this may last only six months or lifelong. Latent TB infection (LTBI) causes no symptoms, and is not contagious. Usually the only detectable abnormality is a positive tuberculin skin test. The World Health Organization has estimated that close to **two billion people** have LTBI ^2, 3^, of whom approximately 10% will reactivate over their lifetime. Therapy can reduce the likelihood of future active disease, but is inefficient, because the subgroup that will develop disease can not be distinguished from the majority who will not, although some have recognized risk factors (RCT Appendix 1: Table 1) ^18-53^.

**1.1.3 Importance of therapy of latent TB infection (how many are treated?)**

In a recently completed survey, we found that more than $25 million is spent annually in Canada for the diagnosis and treatment of approximately 20,000 persons with latent TB infection in 2006^54^. One survey of 110 US health departments found that 127,996 persons initiated LTBI therapy between 2000-2002 ^55^. Based on this, and a second survey of 37, 857 patients in 244 U.S. health departments, it has been estimated that 290,000- 433,000 persons are treated annually for LTBI in the US ^56^, of whom more than 80% receive Isoniazid daily for 9 months (9INH) ^57^. This data and reports from other countries ^58-60^ indicate that LTBI therapy is a major component of TB control in many high income countries. In low and middle income countries the World Health Organization has recommended LTBI therapy for close contacts, especially children, of patients with smear positive pulmonary TB, and is promoting expansion of use of INH for HIV infected persons with LTBI.

**1.2 The principal research questions**

**Hypothesis**: Therapy of latent TB infection (LTBI) with four months of daily Rifampin (4RIF) will result in cumulative incidence of microbiologically confirmed active TB during 28 months following randomization, that is significantly lower than the cumulative incidence of active TB among participants randomized to nine months of daily isoniazid (9INH).

**Primary objective:** To compare the cumulative incidence during 28 months after randomization, of confirmed active tuberculosis (TB) among all persons randomized (effectiveness, using intention to treat analysis) to 4RIF and 9INH.

**Secondary objectives:**

(i) Compare the cumulative incidence of confirmed active TB among those who took at least 80% of doses of the LTBI treatment to which they were randomized, in less than 120% of the allowed time (i.e. ***efficacy*** ).

(ii) Compare the cumulative incidence of ***probable***, as well as confirmed active TB between patients randomized to the two regimens during 28 months following randomization.

(iii) Compare rates of ***Grades 3&4 adverse events*** during treatment between subjects randomized to the two regimens.

(iv) Compare ***health system costs, and cost-effectiveness*** of the two regimens, in the different sites.

(v) Describe occurrence of ***drug resistance*** (to INH or RIF) among subjects who develop confirmed active TB.

**1.3 Why is a trial needed now?**

**1.3.1 History of preventive therapy (“the INH story”) (see RCT Appendix 1 Tables 2&3)** ^61-73^

In the 1950's and 1960's, a number of large scale placebo-controlled randomized clinical trials of Isoniazid (INH) therapy for LTBI were conducted. In trials where high compliance was achieved, or in subgroup analysis of “completer-compliers” 12 months of INH resulted in risk reductions of 83-93%, relative to placebo ^63, 65, 67^. About 50,000 subjects received INH, yet adverse events, including hepatitis were uncommon, and there were no deaths related to drug induced toxicity in these studies ^61, 62, 64^. As a result of this evidence of efficacy and safety, in 1971 the American Thoracic Society (ATS) issued recommendations strongly encouraging INH therapy of LTBI ^74^. The resultant widespread use of INH was quickly followed by the widespread occurrence of hepatitis - with fatalities^75^ ^69^ ^76^, which, together with apparently contradictory risk benefit analyses ^77-81^, resulted in widespread doubts about the benefits, and revised recommendations for use of INH^82-84^.

Publications in the past decade have reported much lower rates of hepatotoxicity ^70, 72, 73, 85^ and mortality ^70-72^., which may be due to better selection of candidates for therapy, or closer follow-up. Recent analyses have concluded that INH therapy will be of benefit and cost-effective for healthy infected persons without risk factors, and be very beneficial and cost-effective for infected persons at higher risk of disease ^86, 86, 87, 87-91^. However the overall effectiveness of INH remains low – because of physician under-prescription ^92-96^ (fearing serious side-effects), and poor patient compliance (because of the long duration) ^70, 93, 96-102^, and costly^94^– because of the close monitoring required to detect potential serious, even fatal, adverse events.

**1.3.3 Alternates to INH for therapy of LTBI (RCT Appendix 1: Tables 4&5)**

These problems of INH therapy have stimulated substantial interest in the evaluation of shorter regimens for LTBI.

*1.3.3.1 Rifampin and Pyrazinamide (“The RIF-PZA story” – history repeats itself):* Based on initial animal studies ^103^ and randomized trials among HIV infected ^104-106^ the regimen of 2 months daily Rifampin & Pyrazinamide (2RIF-PZA) was recommended in 2000 ^107^. This was soon followed by reports of severe and fatal drug induced hepatitis ^108-111^. In subsequent studies, serious adverse events, particularly hepatitis were significantly higher among patients given 2RIF-PZA than in patients given INH ^58, 112-115^ ^116^ ^55, 112, 115, 117, 118^, despite close monitoring. Interestingly, default rates ^113, 115, 117, 119^ and costs ^113, 117^ of 2RIF-PZA were the same, or higher than 9INH in several studies. As a result this regimen has been almost totally abandoned ^120^.

*1.3.3.2 - Rifampin alone:* The only published randomized controlled trial with a mono-RIF regimen compared 3 months RIF, 6 months INH, 3 months INH-RIF and placebo. Interestingly, the 3RIF regimen had efficacy of 63%, which was superior to all other regimens, and no hepatotoxicity ^33^. 6 months RIF was given to 157 high school contacts^121^, and 49 homeless contacts ^122^ - of INH-resistant cases. In both series 6RIF was well tolerated, with no subsequent case of active TB. Further evidence of the high efficacy, despite much shorter therapy comes from experience with active TB. In trials with head to head comparisons, addition of RIF allowed the total duration of therapy to be halved ^158,167^. Hence, implicit in our study hypothesis is that efficacy of 4RIF is 90% - the same as 9INH. Several observational studies of LTBI therapy provide further evidence of the advantages of 4RIF. In the first, of 1,379 patients on 4RIF, 1 (0.1%) developed hepatitis, and 987 (72%) completed therapy, compared to 12 (2%) with hepatitis and 405 (52%) completion among the 770 who started 9INH ^123^. In the second, 261 subjects initiated 4RIF, of whom 210 (81%) completed therapy, and 8 (3%) developed SAE (no hepatitis), compared to 113 (53%) completing, and 13 (6%) with SAE (3 with hepatitis) of the 213 who started INH ^124^. In a third study, of 749 given 4RIF, 76% completed and 9 (1.2%) developed SAE (3 = 0.4% with hepatotoxicity )^125^. The past problem of higher cost of RIF has been resolved by dramatic price reductions in the international market ^126^. There has been no emergence of INH resistance following INH therapy of LTBI in several large studies ^62^ ^33, 65, 127^, nor emergence of Rifampin resistance following RIF therapy ^33, 122, 123, 128^ except for one case-report of a patient who was very poorly compliant with therapy ^129^. Rifampin containing regimens would also be more cost-effective than 9INH for LTBI treatment in immigrants from countries with high rates of INH resistance ^130^. In summary, mono-RIF therapy can be effective for LTBI, may be more cost-effective^130^, with better completion rates ^131^, and less hepatotoxicity ^33, 121-124^.

*1.3.3.3 INH and Rifampin (INH-RIF):* In the Hong Kong study, the INH-RIF combination was the most toxic, and least effective of the three active regimens ^33^. A large uncontrolled paediatric case series utilizing regimens of 6, then 4, then 3 months of daily INH-RIF, reported few cases of TB among those treated, although community rates were used for comparison^59^. A recent questionnaire survey reported that 3 of 344 paediatric household contacts treated with 3INH-RIF developed active TB – a rate of 8.7/1,000 ^60^ . This was 48% less than the rate of TB among similar subjects who received placebo in earlier trials ^62^. In Uganda, 3 months daily INH-RIF-PZA was less effective than 6INH, with similar completion rates, and 4 times higher SAE ^128^. In Saskatchewan twice weekly directly observed INH-RIF was well tolerated, with higher completion rates, and lower subsequent TB incidence than patients given 12 months INH ^132^. In three recent trials, completion of 3-4 months of INH-RIF was better than 6INH, with similar adverse events and efficacy ^133-135^

*1.3.3.4 INH-Rifapentine (INH-RPT):* RPT is a new Rifamycin with a half life five times longer than Rifampin that can be given once weekly. In a recent randomized trial, 2 of 206 (1%) TST positive household contacts receiving 3 months of directly observed once weekly RPT-INH developed Grade 3 or 4 hepatitis, compared to 20 of 193 (10%) of subjects who received 2RIF-PZA ^136^. Active TB developed in 3 (1.5%) who received 3INH-RPT, compared to one (0.5%) of the 2RIF-PZA group. A large CDC-sponsored trial comparing 3INH-RPT and 9INH is nearing completion. However RPT is an “orphan drug” produced in limited quantities by the manufacturer, that has had poor results in treatment of active TB ^137^. Its utility may be limited because of the need for direct observation - impractical for private providers, and increasing costs.

**1.3.4 Current Canadian**^138^, **and** **American** ^107^ **recommendations for LTBI therapy**

Until 1999, 12INH was the standard of care in North America, although 6INH was considered acceptable, because the superior completion rate was considered to offset its lower efficacy ^139^. However, based on an analysis by Comstock ^140^, both the ATS and CTS published revised guidelines in 2000, recommending that 9INH should be the standard of care for LTBI therapy, given its 90% efficacy ^140^. 6INH, 2RIF-PZA, and 4RIF were recommended as alternatives.

**1.4 Results from systematic reviews, and meta-analyses**

Several extensive reviews ^62^ and meta-analyses of trials in HIV infected ^141^ and uninfected ^142^ persons have concluded that 6-12 months INH has significantly better efficacy than placebo – in the populations we plan to study. A recent meta-analysis of 5 trials involving a total of 1926 adults randomized to 3INH-RIF or INH concluded that the rate of active TB was similar (4.2% vs. 4.1%) as was the rate of SAE (4.9% vs. 4.8%) ^143^. However 83% of subjects received 6INH, which has efficacy of only 40-70% ^33, 61, 65, 68, 104, 128^. Meta-analyses of 2RIF-PZA have been published ^118^ but this regimen has been abandoned. There are no published meta-analyses of 4RIF, as there are no trials with 4RIF, and only 1 trial with 3RIF ^33^.

**1.4.1 Summary of current evidence**

Each year, more than 20,000 persons in Canada ^54^, and at least 300,000 persons in the US ^144^ initiate LTBI therapy. Over 80% are prescribed 9INH ^57^, which is considered the standard of care ^107, 138^, but is lengthy, costly, may cause serious adverse events, and has poor completion ^70, 93, 96, 98-102^. Of the available recommended alternative regimens, 6INH has efficacy of only 40-70%, and similar risk of adverse events, The 2RIF-PZA regimen was enthusiastically adopted, but then abandoned due to unacceptable toxicity ^108, 109, 112, 117, 145^. 3INH-RIF appears to have similar efficacy as 6INH, but greater toxicity. This leaves 4RIF, for which there is limited efficacy data, but consistent evidence that safety and compliance are better than with 9INH. With CIHR funding we have completed two trials to compare the 4RIF regimen with 9INH (Section 2.19, and manuscripts in Research Module Appendix). In these and other studies, 4RIF had better compliance and completion rates ^123, 124, 131^, lower costs ^146, 147^, and better safety ^123, 146^, particularly less hepatotoxicity ^148^ - the most serious complication with INH.

**1.5**  **How will the results of the trial be used?**

TB is a major global pandemic, and persists in Canada among impoverished and marginalized groups such as urban poor and aboriginal Canadians, as well as immigrants and refugees. Treatment of LTBI has individual and public health benefits, but the current standard of care - 9INH - has serious side effects, and the length of therapy increases costs, yet reduces compliance ^93, 97-99, 149^, and thereby effectiveness. The benefits of a shorter, cheaper, and safer treatment for LTBI would be substantial ^130, 150, 151^. To date, we and others have found that 4RIF has significantly better completion rates ^123, 131^, lower costs ^123, 146^, and better safety ^123, 146^. The proposed study will be the largest trial to evaluate mono-RIF therapy for LTBI, and the first trial to evaluate the currently recommended 4 month RIF regimen. Thus, our proposed study will provide urgently needed data ^152, 153^ on effectiveness and efficacy of this already recommended (and utilized) alternative LTBI therapy. Involvement of international as well as sites across Canada will provide valuable information on feasibility, tolerability, safety, costs, and effectiveness of 4RIF in different settings and patient populations - enhancing the potential applicability of results.

**1.6 Risks for the trial participants**

Available evidence, from *millions* of patients treated for active TB with RIF in combination with other drugs ^154, 155^, suggests that RIF is well tolerated and safe. Hepatitis is the most important and potentially fatal complication of INH and RIF-PZA therapy of LTBI. On the other hand, mono-therapy with RIF has been associated with very low rates of hepatitis – in Phase 1&2 ^131, 148^, and elsewhere ^33, 121-124^ (RCT Appendix 1 - Table 5). Nevertheless, in view of the 2RIF-PZA experience, where initial trials suggested excellent safety, but unacceptable toxicity was seen with introduction into routine practice, it is prudent to continue to closely monitor the safety of 4RIF. This will be done by independent review of all possible Grade 3 or 4 SAE, plus periodic interim analyses of safety during the trial. Patients will be carefully questioned regarding concomitant medications, to identify potential drug interactions, and will be excluded if these can not be managed easily. Mono-therapy of patients with unrecognized active TB, of particular concern in HIV infected patients, may lead to drug resistance, which has serious implications for treatment^152, 153^. In all settings we will ensure that study participants have access to all necessary investigations to exclude active TB, including cultures. In some settings this means budgetary allocations to pay for these investigations if necessary. In the long term, 4RIF will not be useful in resource-limited settings if extensive investigations are needed before starting therapy. Hence, we will develop and evaluate low cost diagnostic algorithms to identify candidates for LTBI therapy likely to have active TB, for use after the trial (RCT Appendix 5). Initial or primary drug resistance can render LTBI therapy ineffective ^127^. However rates of RIF resistance rates are very low and much lower than INH resistance in all participating countries (RCT Appendix 1 - Table 6) ^156^, ^157^, enhancing the rationale and ethical acceptability of evaluating 4RIF. INH is recommended unless prevalence of primary drug resistance exceeds 50% ^107, 158^ - clearly not the case in any country, or if the subject is a contact of INH resistant cases INH ^107, 138^ – an exclusion criteria for this trial. Finally, subjects will be followed two full years after completing 4RIF to ensure early detection of active TB if this therapy fails.

**SECTION 2 THE PROPOSED TRIAL**

**2.1 Study design**

We propose a multi-centre randomized 2-arm positive control open-label clinical trial. Patients prescribed standard therapy for LTBI (i.e. 9 months INH) will be approached to participate before they begin therapy. After providing informed consent, subjects will be registered using a web-based system, accessible at all times. This system will verify eligibility and perform immediate on-line randomization, using a computer generated random sequence, in equal numbers to 4RIF or 9INH - both daily and self administered. Randomization will be stratified by site and in blocks of variable length. The primary end-point will be the occurrence of microbiologically confirmed active TB. Secondary end-points include occurrence of confirmed and probable active TB, Grade 3 or 4 adverse events (SAE), drug resistant active TB, and health system costs. All primary outcomes will be reviewed by an independent 3-member clinical review panel, and all SAE will be reviewed by a different independent 3-member panel. Both panels will be blinded to the study drug, and patient identity.

**2.1.1 Rationale for an open label trial**

The most important departure from the usual methodology of a randomized controlled trial will be the absence of blinding. This is justified by the primary objective to compare the effectiveness of the two regimens which will primarily be determined by the completion rate of therapy. The 9INH regimen has efficacy of approximately 90%^140^ if taken fully - making it virtually impossible to demonstrate superior efficacy with 4RIF. However in routine practice fewer than 50% complete INH therapy ^70, 93, 96-102^, reducing effectiveness to less than 50%. On the other hand, in Phases 1^131^ and 2^148^, and in observational studies ^123, 124^ treatment completion rates with 4RIF have been 20-30% higher than with 9INH – similar to trials comparing different durations of the same drugs, in which shorter duration consistently resulted in superior completion ^65, 159, 160^. The fundamental rationale for this proposal and the basis for the sample size calculations is that the shorter duration makes improved effectiveness plausible, and detectable. To conduct a fully double blind study, patients assigned 4RIF would take an additional 5 months of similarly coloured and shaped placebo. This would eliminate the most important advantage of 4RIF, and under-estimate its effectiveness. As well, RIF produces reddish discoloration of the urine, so patients assigned to 9INH would take Iron oxide capsules for 9 months. This would render the interventions even less like routine practice, likely reduce compliance even further, and potentially reduce the effectiveness of both regimens to ethically unacceptable levels. Finally, the proposed design is consistent with all other non-placebo controlled trials of LTBI regimens of unequal length – which were all open label, and used microbiologically confirmed active TB as the primary end-point^61, 66, 105, 117, 161^ ^65, 115, 115, 136^, as proposed here. The only published double blinded trials of LTBI therapy used placebo regimens of equal length^62-64^ ^104^ ^33, 68, 162^. However, given the consistent benefit of INH in these trials a placebo controlled trial would not be considered ethical.

**2.1.2 Rationale for the duration of follow-up**

The total duration of follow-up will be 28 months. This offers the best trade-off between the greater potential losses and higher costs associated with longer follow-up, and maximizing detection of incident cases of active TB, since disease risk is highest in the first 2-3 years after tuberculin conversion^42, 43^, detection of contacts^62^, diagnosis of inactive TB ^65^, or following migration from high to low incidence countries ^163^ ^164^. Post-treatment follow-up will be unequal between subjects who complete therapy – 24 months after 4RIF, compared to 19 months after 9INH. This is justified because: (i) We are most concerned about therapeutic failure with 4RIF, given its unknown efficacy. Hence longer follow-up after therapy will enhance our chances to detect failure in compliant subjects – an outcome of key interest. (ii) On the other hand, given the well documented high efficacy of 9INH, therapeutic failure is unlikely in subjects who complete 9INH, so almost all events of active TB will occur in those who drop-out of INH therapy. Since most drop-outs occur early in therapy ^148^, length of follow-up after stopping treatment will actually be similar in those actually at risk to develop active TB; (iii) If anything, this approach will favour 9INH slightly, because patients who completed 4RIF will have 5 more months to be re-infected - plausible in high-incidence settings.

**2.2 Interventions**

The standard therapy will be daily self-administered INH, 5 mg/kg/day (max=300mg/day) for 9 months (9INH). Dosage will be adjusted if weight is less than 42 kg at 200mg/day. As currently recommended ^107, 138, 158^ vitamin B6 (pyridoxine) will be given with INH only to patients with risk factors for neuropathy - malnutrition, alcoholism, diabetes, or renal insufficiency or HIV positive. The experimental arm will be daily self-administered RIF, 10 mg/kg/day (max=600mg/day) for 4 months (4RIF). Dosage will be adjusted if weight is 36-49 kg at 450 mg/day or at 300 mg/day for weight of 35 kg and less^107, 138, 165^.

**2.2.1 Rationale for the interventions**

We have selected 9INH as the standard regimen because: (i) in current guidelines this is the preferred regimen for LTBI^107, 138, 158^; (ii) It is the current standard of practice in North America ^97^, used in 84% of patients treated for LTBI ^57^; and, (iii) INH has consistently had significantly better efficacy than placebo in all trials with study populations similar to the populations we plan to study ^62, 141, 142^; (iv) in systematic reviews ^62^, and meta-analyses ^141, 142^ 9INH has efficacy of as much as 90% ^140^ if taken properly. We have selected 4RIF as the alternative regimen because this is one of three alternates recommended by the CTS and ATS ^107, 138^. Of the two others, 6 months INH had efficacy of 40-69% in several trials ^33, 61, 65, 68, 104, 128^, and the 2RIF-PZA regimen has unacceptable hepatotoxicity ^109, 112, 117, 145^. In our earlier trials and observational studies, 4RIF has had lower toxicity ^123, 124, 148^, lower cost ^147^and better completion ^123, 124, 131^ than 9INH. There is evidence to suggest the efficacy of 4RIF is as high as that of 9INH, but efficacy has not been adequately assessed.

**2.3 Procedures of recruitment, registration, and randomization**

**2.3.1 Recruitment**:

Patients prescribed 9INH therapy will be asked, by their TB care provider, for permission to be contacted by study personnel. Those that agree will be approached to verify eligibility, and obtain signed informed consent. (See Research module Appendix 1 – informed consent used in Phase 2)

**2.3.2 Registration**

Once subjects have provided informed consent, research staff will access the registration and randomization website via the Internet. This computer based program will be available 24 hours/day and 365 days/year.

**2.3.3 Randomization**

We propose central, web-based randomization by a computer generated random number producing algorithm, in blocks of varying length (2 – 8 subjects), stratified by centre. Patients who are household contacts of active cases will be allocated to the same regimen as the first member of that household. Because study personnel may recruit more contacts to one arm if they believe it superior, all contacts in one household must be recruited and randomized at once. If additional household members are enrolled at a later date, they will be randomized separately.

**2.3.4 Rationale for stratification by centre**

The characteristics of the patient populations, their risk of TB, as well as physicians’ usual practice – in starting LTBI therapy, or stopping it because of patient intolerance, will vary considerably by centre. Randomization stratified by site should ensure that these and other potential sources of bias by centre are balanced. As well this will ensure that balance is maintained if one or more centres stop enrolling ^166^, as occurred in Phase 2.

**2.3.5 Maintaining Confidentiality**

The Web-based registration system is non-nominal. At the time of registration, all patients are assigned a unique study ID number. This will be used to label forms, clinical data, and all case report forms. All patient information sent to the data coordinating centre, or reviewed by independent panels, will contain only the study ID. All contact information including names, address, telephone numbers, other contact persons, and full date of birth will be stored at each site, in a secure location, and safeguarded by the site PI. In Canada, lists of participants’ names will be forwarded by registered courier to Provincial health authorities to assess if they develop active TB, following procedures approved by provincial privacy commissions. At the international sites the patients’ names will be cross-checked against reported TB cases at State, or National level, using appropriate safeguards for patient confidentiality. Protocols will be written for protection of confidentiality; these will be a focus of training and ongoing monitoring of all sites.

**2.4 Protection against bias**

**2.4.1 Preventing bias in ascertainment of the primary outcome of active TB**

Given the absence of blinding, it is important to control for potential bias in ascertaining the primary study outcome. To reduce bias in finding or diagnosing cases, the questionnaire used for follow-up, and the clinical evaluation including diagnostic procedures for active TB will be standardized. The diagnostic process may vary if subjects seek care from non-study providers, but this should not be biased by the LTBI regimen. Each member of the clinical panel will review all available clinical evidence including X-rays, pathology, and microbiology reports, while blinded to subjects’ identities, LTBI therapy, and opinions of the other panel members. Each will independently determine if the definition of confirmed active TB is met – ie positive cultures or nucleic acid amplification test for *M. Tuberculosis*, or positive biopsy (see Section 2.8.1 for details). These objective microbiological criteria should not be influenced by patient, nor provider bias, and are the standard outcome for all LTBI trials ^33, 61, 65, 68, 104, 128^.

**2.4.2 Preventing bias in ascertaining secondary outcomes**

The secondary outcome of probable TB, defined as compatible clinical and radiographic features will be judged by the same 3-member clinical review panel, using the same approach as above. All possible Grade 3 or 4 SAE will be investigated using standardized protocols, and graded according to ATS guidelines for hepato-toxicity ^155^, or the National Cancer Institute Common Terminology Criteria for Adverse Events v2.0 (at <http://ctep.info.nih.gov/reporting/ctc.html>) for all others. Grading, and attribution of cause, will be performed by three independent reviewers blinded to the identity of patients, study drug, and opinions of the other reviewers. We will ascertain costs based on visits, tests and other health services – all of which are quite objective. To further minimize bias, the laboratory technicians who perform cultures and drug sensitivity testing, and the cost-effectiveness analyst will be blinded to study regimen.

**2.5 Inclusion and exclusion criteria**

**2.5.1 Inclusion criteria**

Adults (age > 18) with documented positive TST as defined below and prescribed 9INH for LTBI, following authoritative recommendations ^107, 138^:

**Note:** In the absence of a TST test, a positive QFT (or T-Spot) (according to manufacturers recommendations) (see screening, recruitment and randomisation procedures) is equivalent to a TST of 10 mm.

1. HIV positive , OR to start TNFa inhibitors, OR on transplant anti-rejection medications.(TST ≥5 mm or QFT +)

1. Close contact: >4 hours contact per week, for > 1 week with person with active pulmonary TB. (TST >5 mm or QFT +)
2. Apical/upper lobe fibronodular disease with area >2cm^2^ (shown in RCT Quick Guide) (TST >5 mmor QFT +)
3. Documented tuberculin conversion within two years. (Increase ≥ 6 mm, with subsequent TST >10 mm or QFT +)
4. Diabetes, renal failure, or immuno-compromised from medical condition or therapy (TST >10 mm or QFT +)
5. Casual contact: contact of <4 hours/week, with a person with smear positive pulmonary TB. (TST >10 mm or QFT +)
6. Tuberculin conversion within 2-5 years. (Increase of 10 mm or more with subsequent TST >10 mm or QFT +)
7. Have (1) **TWO** of the following  **four** factors if TST = 10-14mm or QFT +,

**OR** (2) **ONE** factor if TST >15mm:

- 1. Arrival in Canada, Australia, or Saudi Arabia in the past 2 years from countries with WHO estimated incidence greater than 100 per 100,000 (these are listed in the RCT Procedure guide - Appendix 5)
  2. BMI <19 (BMI calculations, see RCT Procedure guide);
  3. Any abnormality on chest x-ray compatible with past-TB infection e.g. calcified granuloma, or hilar lymph nodes, costo-phrenic angle blunting - other than fibronodular disease above.
  4. Cigarette smoking (at least a half pack per day) currently.

In the low and middle-income countries, LTBI therapy will usually be offered only to patients in categories 1& 2^158^, because of resource limitations, resulting in substantial differences in risk of active TB among untreated patients at the different sites. Balanced allocation of risk groups to the two study arms will be maintained by site-stratified randomization.

**2.5.2 Exclusion criteria**

1. Patients who were contacts of TB cases known to be resistant to INH, RIF, or both (i.e. MDR)^107, 138^,^127^.

2. Known HIV-infected individuals on anti-retroviral agents whose efficacy would be substantially reduced by Rifampin, unless therapy can safely be changed to agents not affected by Rifampin (listed in RCT quick guide - Appendix 5).

3. Pregnant women - Rifampin and INH are considered safe in pregnancy ^138, 167^, but therapy is usually deferred until 2-3 months post-partum to avoid fetal risk and the potential for increased hepato-toxicity immediately post partum ^168^.

4. Patient on any medication with clinically important drug interactions with INH or RIF, which their physician believes would make either arm contra-indicated. An updated list of clinically important drug interactions is in the RCT Quick Guide (Appendix 5). This includes women taking hormonal contraceptives who will not take alternative contraception.

5. History of allergy/hypersensitivity to Isoniazid or to Rifampin, Rifabutin or Rifapentine.

6. Active TB. Patients initially suspected to have active TB can be randomized once this has been excluded.

7. Persons who have already started LTBI therapy.

**2.5.3 Rationale for exclusion criteria**

We are interested in the real world application of Rifampin, and so will include persons potentially at risk for non-completion or for adverse events. To obtain a realistic estimate of safety and tolerability, no patient will be excluded on the basis of age nor history of TB therapy, liver disease, alcohol use, or other medication use (except as specified above). If the treating physician prescribed LTBI therapy, they must have concluded the benefits of therapy outweighed the risks. Similarly, to obtain a realistic estimate of effectiveness, patients at risk of non-completion (homeless, alcoholic, and drug use) will be included. An additional reason for these inclusion criteria is to enhance comparability with earlier trials ^169^ - which involved subjects who were older^33, 65, 161^, HIV infected^68, 104^, or had other co-morbidities^62^.

**2.6 Duration of treatment**: (see section 2.2 – Interventions)

**2.7 Follow-up and data gathering**

**2.7.1 Initial evaluation**

The initial evaluation, completed by study personnel, will include demographic information, past medical history, reasons for LTBI therapy, tuberculin reactions, chest X-ray findings, other investigations, and HIV status if known. In contrast to patients with active TB, HIV testing of LTBI candidates is not considered essential for patient care ^107, 138, 158^. Hence HIV testing will be offered to all subjects, with appropriate counselling, although it will not be a required test for inclusion in the study. The need for other investigations is at the discretion of the provider, except for subjects with chest X-ray abnormalities with lesions > 2cm^2^, or close contacts with symptoms or X-ray abnormalities, in whom sputum specimens must be sent for AFB smear and culture ^107, 138^. If investigations to exclude active TB are performed, patients can not be enrolled until results are available. We have developed a draft algorithm to identify and investigate subjects with possible active TB (RCT Appendix 3). This will be evaluated and validated to facilitate the use of LTBI therapy in resource-limited settings after the trial is completed.

**2.7.2 Follow-up during treatment**

We wish to ascertain treatment effectiveness under routine programme conditions. Therefore, follow up will be in line with standard practice, and conducted by the initial treating physicians and TB clinic staff, meaning that visits will be monthly for the first 2 months, then every 2 months (minimum) thereafter for both treatment regimens. Blood count (CBC), and liver transaminases will be checked pre-treatment and at the first follow-up visit. Patients will be encouraged to call, or see their TB therapy provider or TB clinic staff, if they develop any new symptoms. Concomitant treatment is a potential problem with un-blinded therapy ^166^ but given the specificity of anti-TB drugs it is highly unlikely that any such therapy would be given, unless active TB was diagnosed. To monitor drug interaction, for medications whose drug levels can be monitored, these will be measured at 0, 2, 4 and 8 weeks, along with any related dosage changes. For anti-coagulants, anti-diabetics, anti-hypertensives or lipid lowering agents the clinical end-points will be monitored - at 0, 2, 4 and 8 weeks of therapy, plus related dosage changes.

**2.7.3 Post treatment follow-up**

After treatment is finished (or discontinued) post-treatment follow-up by telephone, or visit – at the subject’s home, or at the health facility will begin. Follow-up will be every 3 months until 28 months post randomization on the date corresponding to 3 monthly intervals after randomization. Follow-up frequency is because of experience gained with Phase 2 participants – frequent contacts are needed, even if brief, to enhance retention, because so many move, change telephones, or lose interest. Our objective is to achieve a drop-out rate less than 10%.

**2.7.4 Maintaining high quality data:**

*2.7.4.1 Training:* At the start, we will hold a two day training course for all investigators and research coordinators (see Appendix 7- Time-Table) to review eligibility criteria, ethics, consent procedures, protection of confidentiality, registration, randomization, reporting of serious adverse events, and follow-up procedures during and after treatment. Particular attention will be paid to procedures to minimize drop-outs, investigation and management of SAE during therapy, and ascertainment of active TB. Staff at new sites will receive additional intensive training at the study coordinating centre

*2.7.4.2 Supervision:* The PI will visit all new sites within three months after beginning randomization, and all sites annually. The study coordinator will visit new sites at the time of trial initiation, all sites every three months in year 1 and every six months thereafter. We will verify that patients are approached, informed, and provide consent correctly. We will ascertain that documentation of IRB correspondence and consents are complete, confidentiality is protected, and that data entered in databases, or using the Web-based programmes, corresponds to source documents. Verbal feedback will be provided to staff immediately, and written reports will be reviewed with the site investigators and staff, and the principal investigator.

*2.7.4.3 External Audit:* For all Canadian sites, we plan two independent audits – the first 6-9 months after starting enrolment, and the second 18 months later. Both will be performed by a professional clinical research associate experienced in auditing pharmaceutical sponsored trials. For budgetary reasons, the professional auditor will make only one visit to each international site - one year after beginning randomization. The PI will make the second audit visit 12 months later.

*2.7.4.4 Electronic databases - web based and local***:** The web based initial registration incorporates all information from the initial case report form. All SAE and active TB will also be reported using non-nominal web-based forms. To collect all other treatment phase, and post-treatment follow-up information, site staff will use local databases. The data will then be transferred –in non-nominal form- to the coordinating centre. All web-based forms and randomization software, as well as local databases were developed in Phase 2, except for reporting the primary outcome of active TB - which will be added.

**2.8 The primary and secondary outcomes:**

**2.8.1 Definition of the primary outcome**

Confirmed active TB during 28 months after randomization will be defined as a positive culture for *M. tuberculosis*, positive Nucleic acid amplification test for M TB complex, or caseating granulomas in a biopsy from any site. Positive AFB smears will be considered false positive if cultures are negative, but will be considered confirmatory, if cultures failed (for example if contamination or other technical problem occurs).

**2.8.2 Definition of the secondary outcome of probable active TB**

Probable active TB during 28 months after randomization will be defined as a compatible abnormal chest X-ray plus clinical symptoms, which improve following treatment for active TB, as judged on blinded review by a majority of the independent clinical review panel members.

**2.8.3 Other secondary outcomes**

See below for compliance, serious adverse events, costs, and drug resistance.

**2.9 Measuring the primary and secondary outcomes**

**2.9.1 The primary outcome of active TB**

Our primary method to detect active TB will be active follow-up. Participants will be instructed to contact study personnel if symptoms suggestive of active TB arise – during, or after completion of therapy. During LTBI therapy, subjects will be questioned at each follow-up visit for symptoms suggestive of active TB. After therapy has been completed they will be contacted every three months by study personnel until 28 months after randomization. This will be done primarily by telephone in Canadian and Australian sites, by direct patient visits in Brazil and Africa, and both methods in Korea and Saudi Arabia. At each contact, standard questions (listed in RCT Procedures – Appendix 5) will be asked about current symptoms, and if they were diagnosed with TB since last contacted. Any patients with symptoms suggestive of active TB will be evaluated promptly by study personnel following a standardized protocol, including X-rays, sputum AFB smears and cultures. If subjects are diagnosed with active TB elsewhere, information will be collected regarding date of diagnosis, date and type of treatment, treating physician, and health facility. Permission will be sought to obtain clinical, laboratory, and treatment information, and copies of relevant X-rays from the treating physician.

*2.9.1.2 Verifying the diagnosis of active TB*: An independent 3-member clinical panel will review X-rays, and all clinical and lab information. All members are Montreal-based chest specialists with more than 20 years experience, including many patients with active and latent TB (see Research Module Appendix 1); none are co-investigators. Panel members will independently diagnose each case as confirmed, probable, or unlikely TB, as defined above, blinded to patient identity, LTBI regimen, and opinion of treating physician or other panel members. Differences will be resolved by consensus.

*2.9.1.3 Assessing completeness of ascertainment of active TB****:*** To verify the completeness of ascertainment of active TB with our proposed method of follow-up, we will send a list of all randomized subjects to public health officials responsible for the TB registry in each jurisdiction. They will match subjects names with nominal registries held at provincial, state, or national levels. This is included in the consents for Phases 2, and 3 (Research Module Appendix 1). This will allow us to ascertain any under-estimate of the primary outcome, although patients with clinically diagnosed TB, and those who developed TB after leaving the province/state where enrolled may be missed. We will use the same verification procedure for the international sites. In Canada, provincial registries are almost 100% complete for microbiologically confirmed cases, but the completeness, accuracy and comparability of the TB registries at the international sites are unknown. However any possible differences in detection between sites should not bias the comparison of regimens, given the stratified randomization by site.

For the Canadian sites we will ascertain outcomes of consenting participants who were lost to follow-up, through the provincial health administrative databases. We will forward the names of these subjects, to the provincial health authorities 28 months after randomization, to verify whether they have died (if so, date of death), or moved out of province (if so, when). This will enable us to ascertain outcomes of such subjects, and to examine potential bias due to these drop-outs.

**2.9.2 The secondary outcome of compliance during treatment**

Compliance with treatment is an important modifier of treatment effect, and must be measured to perform the planned efficacy analysis among the sub-groups who take therapy per protocol. In Phases 1 & 2 we used the Medication Event Monitoring System (MEMS), to record pill taking behaviour. However we plan to use pill counts as our primary method of assessing compliance because: (i) pill counting is inexpensive, and feasible for all programmes – hence results can be more easily reproduced elsewhere; (ii) in Phase 2 pill counts were highly concordant with the MEMS records (iii) the MEMS is very expensive, and so is unlikely to be adopted in practice – even in North America; (iv) in a previous trial, pill counting had excellent predictive value for risk reduction ^65^.

**2.9.3 The secondary outcome of serious adverse events (SAE)**

At each follow up visit patients will be questioned and examined for evidence of adverse events. Prior to beginning therapy and at the first follow-up visit, CBC, and liver transaminases (AST and ALT) will be tested. As in Phase 2, (see Section 2.19) site investigators will file an initial web-based SAE report if therapy is discontinued because of patients’ symptoms or lab abnormalities. They will be investigated and managed following standardized protocols developed in Phase 2 (RCT Appendix 2), based on our published experience ^170^, recent reviews ^154, 155^ and authoritative guidelines ^138, 155^. Adverse events will be graded as suggested in guidelines by the American Thoracic Society for hepato-toxicity ^155^, and the National Cancer Institute Common Terminology Criteria for Adverse Events v2.0 (at <http://ctep.info.nih.gov/reporting/ctc.html>;). As in Phase 2 we will have an independent 3 member panel (Dr Rick O’Brien, Dr Mike Lauzardo, and Dr Wendy Cronin - letters in Research Module Appendix 1). This panel will review all possible SAE – defined as events that lead to permanent physician discontinuation of study drug, without knowledge of study drug, nor opinions of neither other panel members nor treating physician. They will judge type, severity, and probability of cause of the SAE. Differences will be resolved by consensus. Results of this blinded review by the independent panel will be considered the final diagnosis for type, severity, and relationship to the study drugs. Detection of Grade 3-4 reactions may be greater than in routine practice, but this should occur equally for both arms, and enhance research subjects’ protection. Unreported adverse events cannot be reviewed - leaving room for provider bias. This may occur with minor intolerance, (which will be termed patient non-compliance). However, if the provider stops therapy because of adverse events, this must be reported by investigators, and investigated as above. The anticipated number of participants in each arm will provide substantial power to detect relatively small differences in occurrence of SAE. This should provide the opportunity to detect less common SAE, such as hematologic complications, or ascertain if differences occur within subgroups (e.g. HIV infected). (See also Section 2.18).

**2.9.4 The secondary outcome of drug resistance among cases of active TB**

All positive mycobacterial cultures from subjects who develop active TB within 28 months post randomization will be sent to reference TB laboratories for identification and drug susceptibility testing. All international sites have access to reference TB laboratories, which participate in external quality control programmes with WHO supra-national reference laboratories.

**2.10 Measuring health system costs**

We will gather information on all health services (visits, consults, tests, drugs) used by participants’ during treatment, and if they develop active TB, as an integral part of the local and web-based data-bases. Hence this information will be routinely gathered as part of the trial, as was done in Phase 2. Investigators at each site will provide site specific estimates for values (costs) for all these health services, using methods that we have used in earlier studies ^54, 171-173^. (Details in RCT Appendix 4).

**2.11 Sample size requirements**

Our primary objective is to test whether 4RIF has superior effectiveness compared to 9INH in reducing occurrence of confirmed active TB, based on a planned intention to treat analysis. The number required depends upon the estimated rate of active TB in treated, and untreated patients, and the expected completion rates with 9INH. The expected rate of TB is hard to predict, since we will enrol patients at a wide range of increased risk. In the international sites, where most patients will be contacts (risk over 2 years of 3% ^62^ ^174^ up to 8-10% ^63, 175^), or HIV infected (**annual** risk 3-8% ^20, 21^), cumulative incidence if untreated (or non-compliant) should exceed 5%, but may be only 2% in untreated subjects in Canada. Hence we assume an average cumulative incidence of 3% among untreated. Based on the assumption that 9INH has 90% efficacy if completed ^140^, but only 50% completion rate ^70, 93, 96-102^, and 4IRF efficacy is 90% the required sample size, calculated using Poisson distribution, would be 2,898 participants with full follow-up in each group (see Table S1 below). If re-calculated using the binomial distribution (and [*http://stat.ubc.ca/~rollin/stats/ssize/b2.html*](http://stat.ubc.ca/~rollin/stats/ssize/b2.html)) the sample size required would be 2% to 5% larger. Smaller differences in completion will reduce power, but higher event rates (that are certainly plausible) will substantially increase power.

**Table S1: Sample size required to detect superior effectiveness of 4RIF compared to 9INH**

*(calculated using Poisson distribution ^176^, assuming alpha=0.05 and two-sided tests)*

| **Expected cumulative incidence of TB**  **over 28 months after randomization** | | | **N (per group) to detect difference,**  **with power of:** | | |
| --- | --- | --- | --- | --- | --- |
| **No therapy** | **9INH**  (Completion 55%, Effectiveness 49.5%) | **4RIF**  (Completion 80%, Effectiveness 72%) | **60%** | **70%** | **80%** |
| 3% | 1.49% | 0.84% | 2,648 | 3,337 | 4,243 |
| 4% | 1.98% | 1.12% | 2,013 | 2,537 | 3,226 |
| 5% | 2.48% | 1.4% | 1,598 | 2,013 | 2,560 |
| **No therapy** | **9INH**  (Completion 50%, Effectiveness 45%) | **4RIF**  (Completion 80%, Effectiveness 72%) | **60%** | **70%** | **80%** |
| 3% | 1.65% | 0.84% | 1,809 | 2,279 | 2,898 |
| 4% | 2.20% | 1.12% | 1,357 | 1,709 | 2,173 |
| 5% | 2.75% | 1.40% | 1,086 | 1,368 | 1,739 |

Members of the same household will be randomized to the same regimen. When cluster randomization is used, sample size must be adjusted accordingly because members of the same cluster are no longer independent observations ^177^. Usually, the required sample size is inflated by a factor of *1+(m–1)*ICC*; where *m* is the average cluster size and *ICC* is the intra-cluster correlation coefficient which denotes how similar subjects from the same cluster are with respect to their risk of developing the outcome, compared to those in other clusters ^178^. Given that the cluster sizes will vary (in Phase 2, 20% of subjects were household contacts, of whom 60% were single contacts, and 40%, or 8% of all participants, were in groups of 2-4 persons - with an average number of 2.5 contacts), we adjusted for this via the method described in ^178^. This calculates the design effect as**: *1+[(cv^2^+1)m-1]*ICC*** where ***cv*** is a ratio of the standard deviation of the cluster sizes, ***m*** and ***ICC*** as before. Even assuming an ICC as large as 0.1 (ICC is typically 0.05-0.12 for spouse pairs ^179^), this only results in a design effect of 1.03 - meaning a 3% increase to 2985 per group. Allowing for 10% loss to follow-up the number must be increased to 3283 per arm. Patients will continue to be followed even if they stop therapy themselves (i.e. non-compliance), or their physician stops therapy (e.g. pregnancy or adverse events). Because 847 subjects, enrolled in Phase 2 of this trial, are still being followed for occurrence of active TB using the same methods as proposed here, the total of number of new participants required in this phase can be reduced to 5720.

We have assumed 4RIF efficacy of 90%, based on available evidence. As shown below, if 50% of the 2,898 randomized to each group complete therapy and 28 months follow-up, this would provide more than 90% power, to confirm **non-inferior** efficacy of 4RIF, if the **non-inferiority** margin was 25% - equivalent to a minimum efficacy of 4RIF of 65%. (In other words, we would declare 4RIF **non-inferior** to 9INH if the efficacy of 4RIF was not more than 25% worse than 9INH.) This efficacy has been considered sufficient for authoritative recommendations of 6INH ^107, 138^, which has had efficacy of 40-69% in trials ^33, 61, 65, 68, 104, 128^.

**Table S2: Sample size to assess non-inferiority of 4RIF efficacy**

*(calculated using alpha=0.05 and one-sided test using methods suggested by Blackwelder ^180^)*

| **Expected cumulative incidence of TB** | | **Tolerated difference** | | **Number (per group) required to provide power of** | |
| --- | --- | --- | --- | --- | --- |
| **Untreated** | **9INH** | **Δ (25%)** | **Maximum Event rate with 4RIF** | **80%** | **90%** |
| 3% | 0.3% | 0.75% | 1.05% | 658 | 911 |
| 4% | 0.4% | 1% | 1.4% | 493 | 683 |
| 5% | 0.5% | 1.25% | 1.75% | 394 | 546 |

**2.12 Planned recruitment rate:** (see also Section 2.15)

At the Canadian sites, we anticipate 455 subjects enrolled annually, based on an expectation of similar enrolment at continuing sites, and the addition of Vancouver. The Vancouver site is the largest TB clinic in Canada with more than 800 persons treated for LTBI annually. Given the allocation of the same budget as for Montreal and with 33% more patients, it seems realistic to expect similar recruitment rates as in Montreal. We plan to double the capacity in Brazil by doubling staff and doubling the number of clinics at which patients will be enrolled. We anticipate little change in enrolment in Saudi Arabia. Two new sites will be added in West Africa; both have substantial experience and infrastructure for the conduct of randomized trials of active TB. We anticipate a rapid start-up with an average of 500 enrolments annually from both sites (letter from Dr. Lienhardt - Appendix 1). In Seoul, Korea four University hospitals and the very busy clinic at the Korean Institute of TB (KIT) will be added, under the direction of Dr Woojin Lew (Director of KIT - see letter in Appendix 1). Investigators at KIT were recently awarded a major grant for a 10 year longitudinal study of 3,000 close contacts. It is anticipated that at least 500 contacts with LTBI will be recruited annually at this site. In Australia and Saudi Arabia, the patient volumes are much lower, but addition of these two sites will be valuable because of the high quality TB programmes, and potentially greater generalizability of results to a broader range of settings. In addition both sites will cover all local costs – hence only travel costs for meetings, training, and supervision will be needed (see letters from Dr Marks, and Drs Al Jahdali and Memish - Appendix 1). Dr. Menzies will spend three months during years 1 and 2 in getting the study initiated at all these sites. (This plan is supported by his department Chair - see Dr Eidelman letter in Appendix 1). If all sites enrol a total of 1,950 participants annually (see section 2.15), we should complete enrolment within 3 years, although we have planned recruitment over 3.5 years. And, as in Phase 2, enrolment will be monitored closely, and corrective actions taken promptly - including addition of sub-sites, and, if necessary, shifting operating budgets from under-performing sites.

**2.13 Anticipated problems with compliance and treatment completion**

As discussed in Section 2.3, we expect differences in treatment completion to be the major determinant of differences in effectiveness of the two regimens. Our sample size calculations are based on these expected differences in completion rates, so recruitment will not be further increased to account for non-completion of therapy. We do not anticipate a major problem with “drop-ins” ^166^ during treatment i.e. patients who change therapy and cross over from one arm to the other, as this was seen in less than 1% of subjects randomized in Phase 2. Our planned secondary analysis of efficacy will be based on treatment completion, and assessment of compliance based on pill counting. This measure correlated well with electronic monitoring in Phase 2, and with protective effect in prior trials ^65^.

**2.14 Loss to follow-up**

Our ability to follow all participants successfully for 28 months after randomization will be crucial. Our objective is to have less than 10% drop-outs; higher rates would be of concern, given the anticipated rate of the primary outcome of less than 3%. Loss to follow-up was 17% in Phase 2, although over 97% of subjects had some follow-up (see RCT Appendix 1: Table 7). Based on experience gained in Phase 2, we will ask for home, work, and cellular telephone numbers, plus email addresses of the study participants, at the time of their enrolment. We will also ask for four other contacts – close friends, or relatives living in the same city, or same country, or relatives remaining in their home country for recently arrived immigrants. This approach has been successful, in another ongoing trial of LTBI therapy at the Montreal Chest Institute, in keeping losses to less than 5% of those randomized, during 33 months of follow-up. The initial consent will include subjects’ permission to verify their occurrence of active TB using local nominal TB reporting databases – for all sites, and provincial health administrative data-bases for Canadian sites. This will provide a mechanism (that we have used ^181, 182^) to verify occurrence of active TB passively, and assess under-estimation of the study outcome, due to mortality or migration.

**2.15 Description of study centres, and justification for international sites:**

Selection of the international sites has been based on long-standing collaborative ties ^172, 183-191^, ^192^ between the site investigators and the PI. All involved countries have a substantial burden of TB, and are classified as having intermediate or °high incidence ^2^. Inclusion of these sites strengthens the study for several reasons: (i) TB is a global disease that disproportionately affects low and middle income countries, where it is a very high priority health problem. (ii) LTBI therapy is under-utilized in these countries because it is viewed as impractical – but could have important benefits if therapy was simplified. (iii) Conduct of the trial in these international settings may help to demonstrate the feasibility, and cost-effectiveness of this strategy in these settings. This may enhance uptake of the findings in countries with a substantial TB burden. (iv) The training and conduct of this trial will strengthen capacity for clinical research in each country - an important long-term benefit. (v) These sites enhance the feasibility, and cost-effectiveness of the present trial.

**Participating Canadian and international sites in Phase 3.** (*Four will be new sites. **Brazil will double clinic sites)

|  | Number of patients treated per year (from 2005-2007) | | Eligible per year, (estimated for new sites) | Annual enrolment | |
| --- | --- | --- | --- | --- | --- |
|  | Active TB | LTBI |  | Phase 2 | Projected |
| Montreal Chest | 60-70 | 550-650 | 200 | 140 | 150 |
| Saskatoon | 80-90 | 300 | 50 | 25 | 30 |
| Toronto | 60-70 | 250-300 | 50 | 25 | 50 |
| Edmonton | 70 | 450 | 100 | 60 | 75 |
| Vancouver* | 120 | 800 | 250-300 | -- | 150 |
| Rio de Janeiro, Brazil** | 800 | >1,000 | >500 | 180 | 350 |
| Riyadh, Saudi Arabia | 100 | 300 | 150 | 70 | 70 |
| Korea* | 2560 | na | 2500 | na | 500 |
| Australia* | 300 | 123 | 120 | na | 75 |
| Benin* | >1,000 | na | 500 | na | 250 |
| Guinee* | >1,000 | na | 500 | na | 250 |
| TOTAL | >2,000 | > 4,000 | > 1,600 | 500 | 1950 |

**2.16 Primary data analysis**

The primary study outcome will be the occurrence of microbiologically or histologically confirmed active TB, confirmed by the majority of the 3-member independent clinical review panel. The primary analysis, comparing the rate of occurrence of active TB per patient-year will be performed with the use of an unadjusted Poisson marginal model fitted using generalized estimating equations (GEE) to allow us to take clustering by household into account ^193, 194^. We will assume an exchangeable correlation structure. The log of follow up time will be used as an offset in the regression model, which will allow us to account for differing lengths of follow up time ^194^ If clustering is significant we will calculate a rate ratio from the GEE Poisson regression. But if the effect of clustering is negligible, as anticipated, the proportion of subjects randomized to both treatment groups developing confirmed active TB, and the associated 2-sided 95% confidence interval for the difference, will be estimated, using an incidence density method, expressed as TB events per 1000 person years of follow-up. This will allow us to include information from subjects who are followed for some time before being lost to follow-up.

**2.16.1 Justification**

Our planned primary analysis will be of study groups as randomized, i.e. an intention to treat analysis. This is recommended for superiority studies ^166^ because it provides a more conservative estimate of effect, since patients who did not take treatment per protocol are less likely to gain benefit. Hence differences between two regimens are attenuated making it more difficult to detect superiority.

**2.17 Frequency of Interim analysis and stopping rules**

**2.17.1 Primary outcomes**

Only one interim analysis of the primary outcome will be performed, one year after 33% of patients have been randomized. Further interim analyses, such as one year after 67% of subjects have been randomized, will fall too close to the end of randomization, to have any meaningful impact. We wish to avoid falsely concluding that one regimen has significantly superior effectiveness with this interim analysis – a well known risk ^195, 196^. Hence we will use a threshold of a p value <.001^196^, before concluding that 4RIF is significantly **inferior** to 9INH , and stopping the trial early.

**2.17.2 Serious adverse events (SAE)**

Four months after randomization of 25%, 50%, and 75% of subjects, interim analyses will be performed of SAE. The DSMB will consider stopping the trial early if SAE rates are significantly ***higher*** with 4RIF. To balance the risk of unnecessarily stopping the study with interim analyses ^196^, with the need to ensure patient safety, we will use the method of Pocock ^197^, or an interim stopping level (p value) of 0.018 for each analysis.

**2.18 Planned sub-group and secondary analyses:**

**2.18.1 Active TB in subjects who complete treatment (efficacy)**

Non-completion of therapy is obviously not a random event, and may be associated with characteristics that are associated with risk of disease^166^. Therefore we will compare the characteristics of compliant and noncompliant subjects in each group, and use logistic regression to estimate efficacy, adjusted for important covariates.

**2.18.2 Confirmed and probable active TB**

In secondary analysis we will combine these two outcomes, and use the same methods to compare rates of TB with both regimens, as described above for the primary analysis.

**2.18.3 SAE**

This outcome will be defined as the occurrence of Grade 3 or 4 SAE, diagnosed by the majority of the independent reviewers. Differences in all SAE, and by type of SAE between the two groups will be tested with Chi-squared or Fisher’s exact tests. Sub-group analyses will compare rates of SAE by age, sex and HIV status. Drug interactions with 4RIF are common, and of particular interest since this will be the largest trial with mono-Rifampin therapy. All analysis of SAE and drug interactions will be presented as exploratory, with appropriate caution ^166, 196^. However in view of the very limited published experience with 4RIF, any information about adverse effects, including drug interactions, would be very useful for clinicians.

**2.18.4 Drug resistance among cases of active TB after randomization**

The occurrence of drug resistant TB after LTBI therapy may result from selection of drug resistant mutants during therapy (acquired resistance), or because they were infected with drug resistant organisms (primary resistance). All patients with positive cultures for M Tuberculosis within 28 months after randomization will have drug sensitivity testing. Adequate facilities are available in all sites. If drug resistant tuberculosis is found, patients will be placed on appropriate therapy and followed by the site investigators in collaboration with the local TB programs. We will compare the prevalence of resistance to INH and RIF among study participants who develop active TB. The prevalence of underlying (or primary) INH resistance in the community will be assumed as the prevalence in those who took 4RIF, and of RIF resistance in those on 9INH. Acquired RIF resistance will be taken as the difference in prevalence of RIF resistance between subjects on 4RIF and 9INH. Power to detect significant differences will be limited, unless drug resistance is very common, but descriptive information on any excess resistance would still be useful.

**2.18.5 Cost-effectiveness of the two regimens**

We will have detailed information on all health system activities – including scheduled visits, therapy and tests as part of routine follow-up, unscheduled visits, tests and therapy for adverse events, and diagnostic and therapeutic activities for the cases of active TB that develop. These will be valuated using local costs for all such activities (for details see RCT Appendix 4). We will also have measured incidence of active TB among subjects randomized to the two LTBI regimens. If one arm is cheaper and associated with fewer TB cases then it will be clearly preferable from a cost-effectiveness standpoint. If one arm is more expensive but associated with fewer TB cases then we will calculate the incremental cost per additional TB case prevented by the more effective (but more expensive) regimen. In primary analysis we will use average Canadian costs to valuate all health care activities, but in secondary analyses will perform the same analyses using costs from each site. This will be of particular interest in lower-income countries, where LTBI therapy is considered a low priority ^158, 199^. In sensitivity analyses we will vary the costs for active TB, given that the cases detected in this study will likely be at an early stage, so may underestimate average costs. We will also vary the costs of rifampin, which is extraordinarily expensive in Canada, compared to international prices^126^.

**2.19 Results from Phase 1 and Phase 2**

Six years ago, we initiated a series of studies to evaluate 4RIF as therapy for LTBI. We felt the first essential requirement for 4RIF was that compliance and treatment completion had to be better than with 9INH. Hence these were primary end-points of the first study (Phase 1). Of 116 patients randomized equally to 4RIF or 9INH, 91% completed 4RIF with good compliance compared to 70% of those randomized to 9INH (p<0.001)^131^ ^200^ (See Research Module Appendix: Reprints).

The experience in 2000-2001 with 2RIF-PZA mandated a careful assessment of safety, conducted in Phase 2 - a multi-centre study primarily to compare serious adverse events (SAE) with 4RIF and 9INH. Consenting patients were randomized in equal numbers to 4RIF or 9INH using a web-based patient registration and randomization developed by Dr. Rousseau of the University of Sherbrooke. *To view the registration, randomization, and SAE reporting web-site, use Internet explorer (at least 6.0) to go to:* [http://tbera-demo.crc.chus.qc.ca](http://tbera-demo.crc.chus.qc.ca/)*. Username:* **dmenzies***, password:* **review***; domain:* **rsr** *(until Feb 1 2009). Enter these once when prompted, and then enter the same username and password a second time, when prompted again. This gives administrator rights to view a demonstration database, with fictitious patients.* Randomization was stratified by site in blocks of variable size. Investigators performed a standardized evaluation for each possible SAE. If the study drug was permanently discontinued, all clinical, lab, and follow-up information was reviewed independently by a three member panel, blinded to the study drug, to judge the type, severity and likely relationship to the study drug (Appendix 4).

We experienced more difficulties recruiting patients than anticipated at several Canadian sites. In the first year we intensified training and supervision, and opened a new sub-site. When this was not enough, investigator and CIHR approval was sought to shift budget allocations from low enrolling sites to start two new international sites – in Brazil and Saudi Arabia, ***within the originally awarded total budget***. Enrolment at all sites subsequently increased to average 40 patients per month. We had planned a sample size of 549 per arm to provide 80% power (2 sided test) to demonstrate a significant difference in SAE rate if the true rate with 9INH was 4% and 4RIF was 9%, or was 5% with 9INH and 2% with 4RIF. After 25%, 50% and 75% of planned enrolment, the DSMB reviewed interim analyses – still blinded to the study drugs. In January 2007, the 3^rd^ interim analysis revealed that SAE were significantly fewer in one arm. The DSMB asked to be unblinded, and when they learned that 4RIF had the lower rate of SAE, they recommended discontinuation of enrolment. Of 420 subjects randomized to 4RIF, 7 developed Grade 3-4 adverse events attributed to study therapy by the independent panel, compared to 17 of the 427 on 9INH (Risk difference (4RIF-9INH): -2.3%; [95% Confidence interval: -0.1% to -5%] p=.04). Grade 3-4 hepatitis occurred in 3 taking 4RIF, compared to 16 who started 9INH (-3.1%; [-1% to -5%], p=.003). Grade 1 or 2 adverse events attributed to study drugs were similar in the two arms. Asymptomatic reduction in platelet count and white blood count were significantly more frequent during treatment with 4RIF. Completion rates were 78% with 4RIF and 60% with 9INH (Risk difference: 18% [12% to 24%], p<.001) ^148^. Average health system costs were significantly lower with 4RIF. Incremental cost effectiveness analysis revealed that 4RIF would be cost saving and prevent more cases if efficacy was at least 75%, and would be cost saving if efficacy is more than 65% ^147^.

We believe that a study to assess effectiveness of 4RIF is now justified. This regimen has been demonstrated to have better completion, lower costs, and is safer than 9INH, particularly for hepatotoxicity - the most important and potentially lethal adverse event of INH therapy (and of 2RIF-PZA). In Phase 2, 847 subjects were enrolled, randomized, treated and followed to detect active TB - using the methods proposed here, making the present proposal more cost-effective.

**SECTION 3 TRIAL MANAGEMENT**

**3.1 Day to day running** (See also RCT Appendix 7 - Time-Table)

This is described in Sections 2.3 (recruitment), 2.7 (follow-up/data gathering), and 2.8-2.9 (measuring outcomes).

**3.2 Role of applicants**

Dr Menzies will chair the trial steering committee, supervise the central coordinating staff, liaison with site PI’s, and take primary responsibility for overall data analysis and report writing. Each site PI will supervise all aspects of site trial management (ethics, recruitment, and follow-up), and site-specific staff. To take advantage of members’ expertise, added specific responsibilities will be assigned as described in detail in RCT Appendix 6.

**3.3 Trial steering committee, DSMB, and Clinical review panel**

The trial steering committee will consist of the PI, site investigators, and study manager (Mme Hornby). They will meet by telephone conference every 3 months in the first year, and then every 6 months, to review recruitment, randomization, operational issues, and DSMB reports. This committee will decide on early termination (if recommended by the DSMB), major protocol and/or consent modifications, and budget re-allocations. The membership, responsibilities, and functioning of the DSMB are described in Section 2.9.3, and of the clinical review panel in Section 2.9.1.
